# Global-local least-squares support vector machine (GLocal-LS-SVM)

**DOI:** 10.1371/journal.pone.0285131

**Published:** 2023-04-27

**Authors:** Ahmed Youssef Ali Amer

**Affiliations:** Data Sciences, Janssen Pharmaceutica NV, Beerse, Belgium; Jeonbuk National University, KOREA, REPUBLIC OF

## Abstract

This study introduces the global-local least-squares support vector machine (GLocal-LS-SVM), a novel machine learning algorithm that combines the strengths of localised and global learning. GLocal-LS-SVM addresses the challenges associated with decentralised data sources, large datasets, and input-space-related issues. The algorithm is a double-layer learning approach that employs multiple local LS-SVM models in the first layer and one global LS-SVM model in the second layer. The key idea behind GLocal-LS-SVM is to extract the most informative data points, known as support vectors, from each local region in the input space. Local LS-SVM models are developed for each region to identify the most contributing data points with the highest support values. The local support vectors are then merged at the final layer to form a reduced training set used to train the global model. We evaluated the performance of GLocal-LS-SVM using both synthetic and real-world datasets. Our results demonstrate that GLocal-LS-SVM achieves comparable or superior classification performance compared to standard LS-SVM and state-of-the-art models. In addition, our experiments show that GLocal-LS-SVM outperforms standard LS-SVM in terms of computational efficiency. For instance, on a training dataset of 9, 000 instances, the average training time for GLocal-LS-SVM was only 2% of the time required to train the LS-SVM model while maintaining classification performance. In summary, the GLocal-LS-SVM algorithm offers a promising solution to address the challenges associated with decentralised data sources and large datasets while maintaining high classification performance. Furthermore, its computational efficiency makes it a valuable tool for practical applications in various domains.

## 1 Introduction

One important property inferred from machine learning algorithms, especially support vector machines (SVM), is that not all data points (equally) contribute to the model (i.e. sparseness for SVM). In other words, for a support vector machine model, only support vectors matter as they are the only data points that contribute to the model [1, 2]. However, the only way to determine these support vectors is by developing and optimising the SVM model. Developing and optimising such a model can be computationally expensive for large-size data sets and requires having all the data points in the same data pool to train a global model. Therefore, it would be computationally more efficient if these data points were determined in advance. From the computational complexity perspective, (much) fewer data points are needed to train a general SVM model. Furthermore, handling the challenge of having the training data set in decentralised data pools can be achieved by determining the support vectors locally. As a parallel support vector machine approach, a similar idea was introduced in the study of cascade SVM [3]. In the approach of cascade SVM, the training data is split into multiple subsets; for each subset, a small SVM model is built. The small SVM models determine the support vectors for each subset and are then transferred to the next layer to train SVM models by the extracted support vectors. This approach is implemented on multiple layers, and the final layer comprises one final global SVM model, which is trained by the support vectors from the different subsets and filtered through the cascade layers. The final layer model is evaluated by applying it to the different subsets at the first layer [3].

SVM is known for being computationally expensive due to its reliance on quadratic programming to optimise the model [1]. Therefore, the least-squares support vector machine algorithm (LS-SVM) was developed to lower the computational cost by handling the optimisation problem by solving a set of linear equations [2]. However, LS-SVM lacks the property of sparseness of SVM since all data points contribute with different weights to the LS-SVM model. On the other hand, sparseness can be recalled to the LS-SVM by applying a pruning algorithm to obtain a sparse LS-SVM algorithm [4, 5], especially that the sparse approximation was proven to be equivalent to the standard SVM [6].

Several challenges motivate the introduction and investigation of the proposed algorithm of GLocal-LS-SVM. These challenges are distributed data sources, decentralised machine learning, federated learning and large-size data sets in addition to some input-space-related challenges.

The key property of the GLocal-LS-SVM is the integration between the localised and the global learning approaches. Since the localised learning algorithms have shown many capabilities to handle several input-space challenges such as class imbalance, ambiguity and rare events [7, 8]. On the other hand, localised learning builds a locally efficient model, however, it cannot inform about a model over the whole input space. Furthermore, investigating the whole input space is needed to build the global model. Therefore, the proposed algorithm of the GLocal-LS-SVM uses the localised learning algorithm to capture the local characteristics of the data in the different regions of the input space. In addition, the global learning algorithm is used to merge the extracted information from the localised models to build the global model.

The proposed approach of Glocal-LS-SVM overlaps with three learning approaches: federated learning, deep learning and cascade SVM. Regarding federated learning [9, 10], the overlap is present in developing individual models locally at different data sources and then merging these individual models into one general model. This general model is built based on collecting the parameters of these individual models and synthesising them into that general model. The common aspect between federated learning and GLocal-LS-SVM is building one general model based on the obtained information from the local models. However, this information is about data points instead of model parameters. For deep learning, the overlap is present in extracting the most informative portion of the data to learn a model. This informative portion is a set of features extracted by consecutive neural network layers for deep learning. However, for our approach, this informative portion is a set of data points extracted by (at least) an extra layer of local learners preceding the global learner.

The third overlapping approach is the cascade SVM, with which the GLocal-LS-SVM approach overlaps in extracting the most informative data points (i.e. support vectors) in parallel from distributed models. These distributed models are small SVM models. However, the GLocal-LS-SVM is based on developing local LS-SVM models. This contrast inherits two significant differences: the first is the lower computational complexity of LS-SVM models. The second difference is the lack of sparseness for LS-SVM, which enables more flexibility in determining the most informative data points proportional to the support values associated with the different data points. This flexibility is absent in standard SVM as the support vectors are determined in a binary way.

Our hypothesis for the GLocal-LS-SVM is to obtain the most informative data points locally that define each region’s essential details. We can build a general model with less computational complexity and comparable error performance through these obtained data points. Moreover, the GLocal-LS-SVM approach enables the development of models locally without collecting all data on one server. In addition to supporting federated learning, distributed and edge machine learning are essential to many artificial intelligence applications, especially those relying on large quantities of data that are generated by distributed data sources [11, 12]. Ultimately, we foresee that parallel computing can handle our proposed approach efficiently.

This article is structured as follows. Section 2 explains the GLocal-LS-SVM algorithm based on the standard SVM and LS-SVM algorithms. Next, GLocal-LS-SVM and LS-SVM Performances are compared in Section 3 using a synthetic dataset. Then, in Section 4, GLocal-LS-SVM and various state-of-the-art classifiers are applied to three real-world datasets, and their performances are compared. Finally, the results are discussed, and the conclusions are presented in section 6.

## 2 GLocal-LS-SVM algorithm

This section introduces the method used to implement the global-local LS-SVM (GLocal-LS-SVM). The essential hypothesis is to build local LS-SVM models scattered over the input space of the training data. These local models act as oracles that inform the support vectors for each region in advance. Therefore, we hypothesise that the data points that act as support vectors for local regions shall be informative enough to act as the general model’s training data set.

The implementation of our approach is based on the sparse least-squares support vector machine algorithm of [5] since the local models and the general model are LS-SVM models. Moreover, a supportive data partitioning algorithm is needed to split the training dataset into local regions. An example of a data partitioning algorithm is the Kmeans clustering algorithm [13] which is used in this study.

### 2.1 Standard support vector machines

SVM’s are presented initially as binary classifiers that assign each data instance $\text{x}\in R^{d}$ to one of two classes described by a class label *y* ∈ {−1, 1} based on the decision boundary that maximises the margin 2/‖**w**‖_2_ between the two classes. The margin is determined by the distance between the decision boundary and the closest data point from each class [1, 2].

Generally, a feature map $\phi :R^{d}\mapsto R^{p}$, where *d* is the number of input space dimensions and *p* is the number of feature space dimensions, is used to transform the geometric boundary between the two classes to a linear boundary *L*: **w**^⊤^ *ϕ*(**x**) + *b* = 0 in feature space, for some weight vector $\text{w}\in R^{p\times 1}$ and b∈R. The class of each instance can then be found by *y* = sgn(**w**^⊤^ *ϕ*(**x**) + *b*), where sgn refers to the sign function. The estimation of the boundary *L* is performed based on a set of training examples **x**_*i*_ (1 ≤ *i* ≤ *N*) with corresponding class labels *y*_*i*_ ∈ {−1, 1}, where *N* is the number of data points. An optimal boundary is found by maximising the margin defined as the smallest distance between *L* and any of the training instances. In particular, one is interested in constants **w** and *b* that minimise a *loss-function* [2]:

$$\begin{array}{c} \underset{\text{w},\;b;\xi }{\text{min}}\frac{1}{2}{\text{w}}^{⊤}\text{w}\;+C\sum_{i=1}^{N}\xi_{i}, \end{array}$$ (1)

and are subject to:

$$\begin{array}{cc} & y_{i}\left({\text{w}}^{⊤}\phi \left({\text{x}}_{i}\right)+b\right)\geq 1-\xi_{i},\;\;i=1,2,\ldots ,N,\\ & \xi_{i}\geq 0,\;\;i=1,2,\ldots ,N. \end{array}$$

By applying the lagrangian method to the problem we get

$$\begin{array}{c} L\left(\text{w},b;\alpha \right)=\frac{1}{2}{\left\|\text{w}\right\|}_{2}^{2}-(\sum_{i=1}^{N}\alpha_{i}\left(y_{i}\left[{\text{w}}^{⊤}\phi \left({\text{x}}_{i}\right)+b\right]-1+\xi_{i}\right)-\sum_{k=1}^{N}\nu_{k}\xi_{k}, \end{array}$$

where *α*_*i*_ ≥ 0 is the Lagrangian multiplier for *i*^*th*^ data point. By solving the optimisation problem

$$\begin{array}{c} \underset{\alpha ,\nu }{\text{max}}\;\underset{\text{w},b,\xi }{\text{min}}\;L\left(\text{w},b,\xi ;\alpha ,\nu \right), \end{array}$$

the following optimisation conditions are obtained:

$$\begin{array}{cc} \frac{\partial L}{\partial \text{w}}=0 & ⟶\text{w}=\sum_{i=1}^{N}\alpha_{i}y_{i}\phi \left({\text{x}}_{i}\right),\\ \frac{\partial L}{\partial b}=0 & ⟶\sum_{i=1}^{N}\alpha_{i}y_{i}=0,\\ \frac{\partial L}{\partial \xi_{k}}=0 & ⟶0\leq C\leq \alpha_{k},\;\;k=1,2,\ldots ,N. \end{array}$$

The resulting classifier in both primal space and dual space are

$$\begin{array}{c} f\left(\text{x}\right)=\text{sgn}\left({\text{w}}^{⊤}\phi \left(x\right)+b\right), \end{array}$$

$$\begin{array}{c} f\left(\text{x}\right)=\text{sgn}(\sum_{i=1}^{\#SV}\alpha_{i}y_{i}\phi {\left({\text{x}}_{i}\right)}^{⊤}\phi \left(x\right)+b). \end{array}$$

The dot product *ϕ*(*x*_*i*_)^⊤^ ***ϕ***(**x**) is computationally expensive, hence, it is replaced with the *kernel* function *k*(*x*_*i*_, **x**), this replacement is known as the *kernel* trick. With the *kernel* trick, there is no need to execute the step of feature map as it is implicitly done by the *kernel* function. Hence, the dual space classifier with the *kernel* trick is

$$\begin{array}{c} f\left(\text{x}\right)=\text{sgn}(\sum_{i=1}^{\#SV}\alpha_{i}y_{i}k\left(x_{i},\text{x}\right)+b). \end{array}$$ (2)

As shown in (2), the classifier in the dual space considers only the support vectors (#SV) instead of the total number of data points *N* since many *α*_*i*_ are zeros leading to sparseness.

### 2.2 Least-squares support vector machine

LS-SVMs are obtained by using a least-squares error loss function [14]:

$$\begin{array}{c} \underset{\text{w},\;b;\;e}{\text{min}}\;\frac{1}{2}{\text{w}}^{⊤}\text{w}\;+\frac{1}{2}\gamma \sum_{i=1}^{N}{e_{i}}^{2}, \end{array}$$ (3)

such that

$$\begin{array}{c} y_{i}\left({\text{w}}^{⊤}\phi \left({\text{x}}_{i}\right)+b\right)=1-e_{i},\;i=1,2,\ldots ,N. \end{array}$$

This optimisation procedure introduces errors *e*_*i*_ such that 1 − *e*_*i*_ is proportional to the signed distance of **x**_*i*_ from the decision boundary. In fact, the non-negative slack variable constraint is removed and the solution of the optimisation problem can be obtained by a set of linear equations, reducing computational effort [14].

Similar to the standard SVM, by applying the lagrangian method to the problem we get

$$\begin{array}{c} L\left(\text{w},b;\alpha \right)=\frac{1}{2}{\left\|\text{w}\right\|}_{2}^{2}-(\sum_{i=1}^{N}\alpha_{i}\left(y_{i}\left[{\text{w}}^{⊤}\phi \left({\text{x}}_{i}\right)+b\right]-1+e_{i}\right), \end{array}$$

where *α*_*i*_ ≥ 0 is the Lagrangian multiplier for *i*^*th*^ data point. By solving the optimisation problem

$$\begin{array}{c} \underset{\alpha }{\text{max}}\;\underset{\text{w},b,e}{\text{min}}\;L\left(\text{w},b,e;\alpha \right), \end{array}$$

the following optimisation conditions are obtained:

$$\begin{array}{cc} \frac{\partial L}{\partial \text{w}}=0 & ⟶\text{w}=\sum_{i=1}^{N}\alpha_{i}y_{i}\phi \left({\text{x}}_{i}\right),\\ \frac{\partial L}{\partial b}=0 & ⟶\sum_{i=1}^{N}\alpha_{i}y_{i}=0,\\ \frac{\partial L}{\partial e_{k}}=0 & ⟶\alpha_{k}=\gamma e_{k},\;\;k=1,2,\ldots ,N. \end{array}$$

The resulting classifier in both primal space and dual space are

$$\begin{array}{c} f\left(\text{x}\right)=\text{sgn}\left({\text{w}}^{⊤}\phi \left(x\right)+b\right), \end{array}$$

$$\begin{array}{c} f\left(\text{x}\right)=\text{sgn}(\sum_{i=1}^{N}\alpha_{i}y_{i}k\left({\text{x}}_{i},\text{x}\right)+b). \end{array}$$

As shown in the third optimisation condition, *α*_*k*_ is nonzero which leads to the lack of sparseness in contrast with standard SVM. Moreover, the support values *α*_*k*_ are proportional to the error variable *e*_*k*_ which reflect the importance of the associated data points **x**_*k*_ and their contribution to the LS-SVM model.

**Fig 1:**
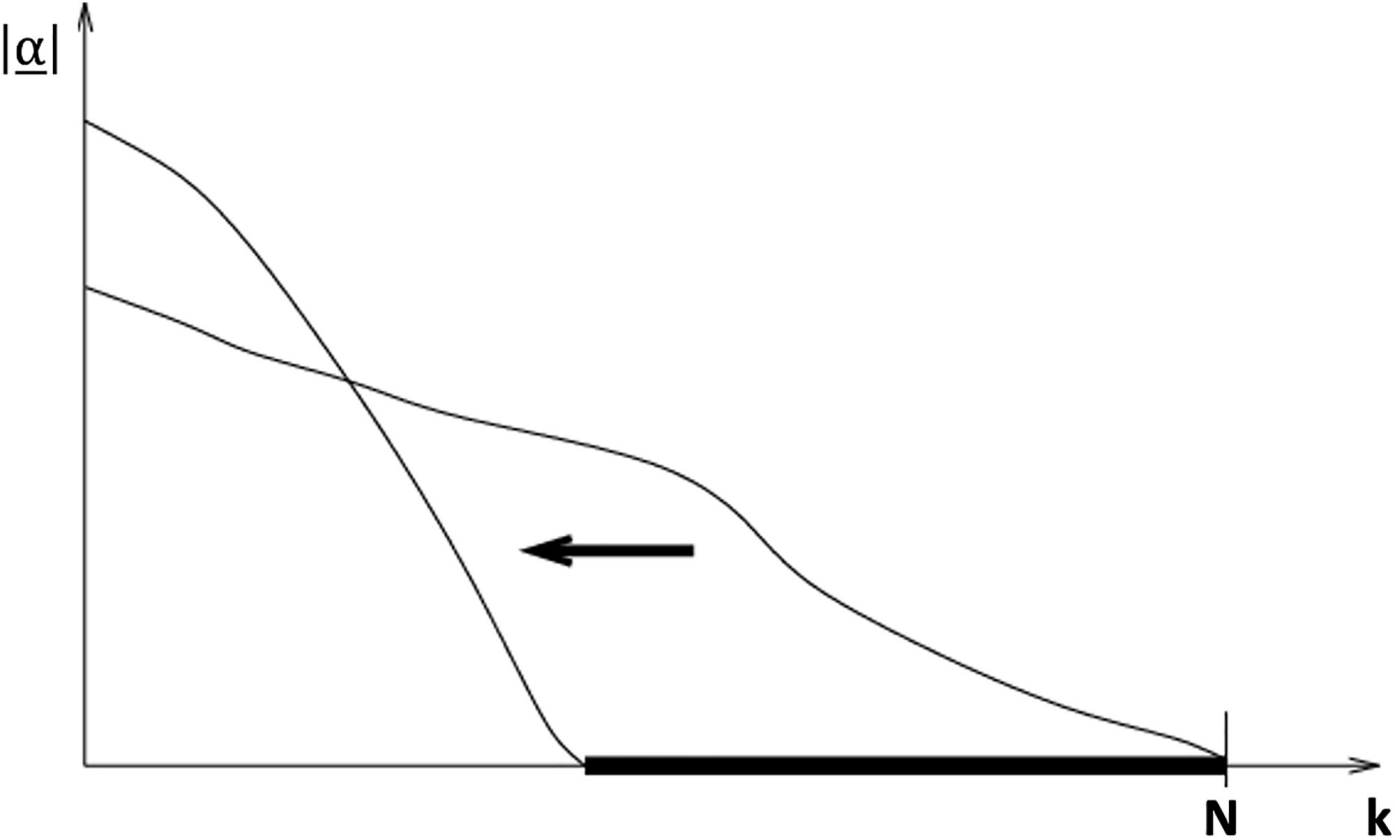


Several studies investigated the possibility to bring back sparseness to LS-SVM [4–6, 15, 16]. In the studies [4, 5, 15], Suykens et al. proposed pruning algorithms to keep only the most relevant data points associated to the highest support values *α* as shown in Fig 1 by eliminating the low-value support values while maintaining the error performance.

Moreover, in the study of [6], Girosi et al. proved the equivalence between the sparse approximation of LS-SVM and the standard SVM from the error performance perspective. Therefore, in the proposed algorithm of GLocal-LS-SVM, the sparse LS-SVM [5] is the used algorithm to develop the local models.

### 2.3 Global-local LS-SVM

As illustrated earlier, for LS-SVM, all data points are considered as support vectors with different weights proportional to their contribution to the model. Therefore, only a portion of the data contributes significantly to the model. This data portion consists of the data points with the highest support values *α*. Furthermore, it is needed to determine this data portion prior to building a general model with a less computationally expensive methodology. We hypothesise that this less expensive methodology is developing a set of smaller local LS-SVM models scattered over the data space. In the case of already distributed data sources, smaller local models can be developed at each data source to extract the local most informative data points.

**Fig 2:**
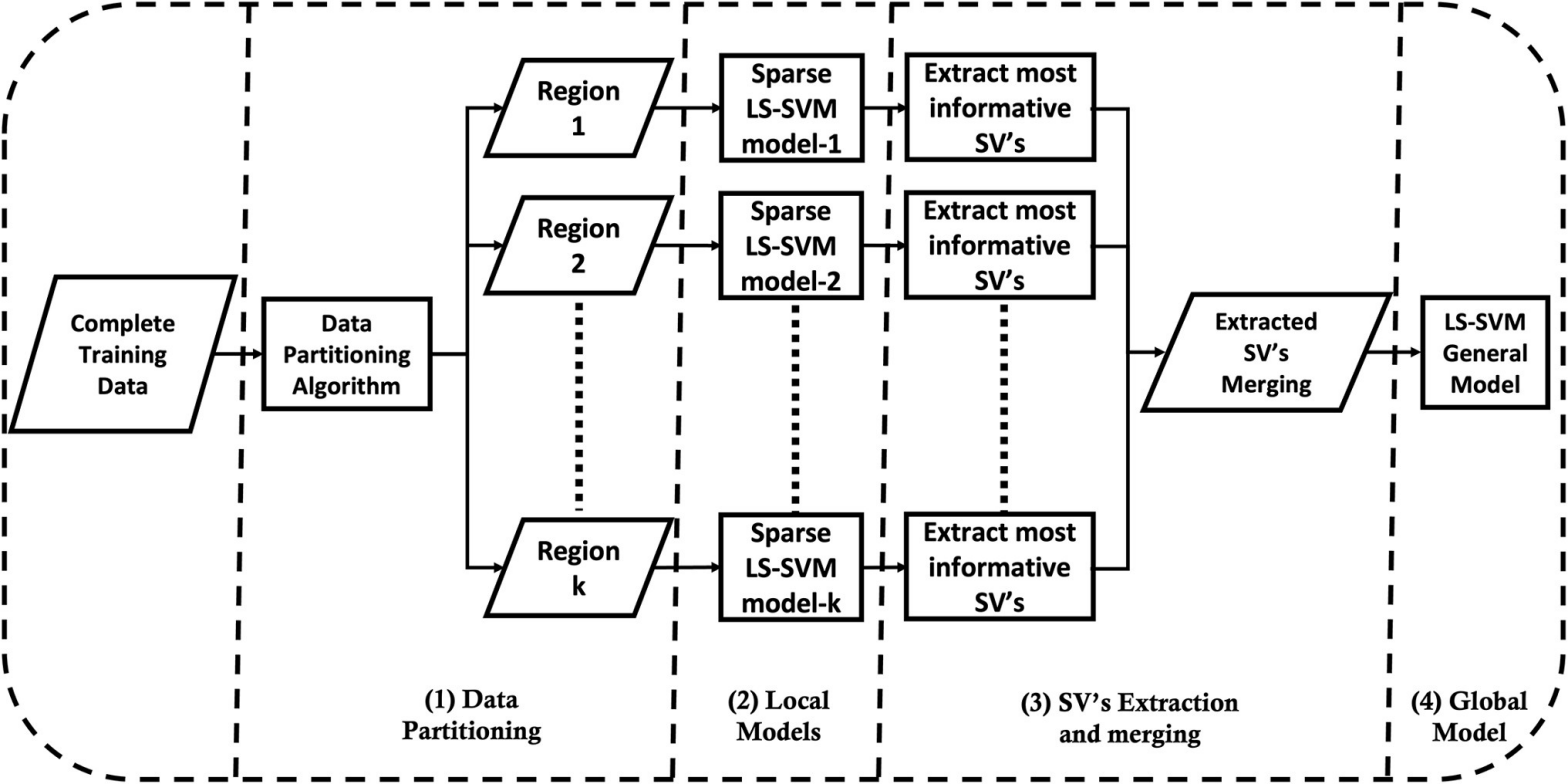


As shown in Fig 2, the implementation of the algorithm follows the steps below:

1. Given a complete training dataset, apply a data partitioning algorithm (e.g. Kmeans clustering) to partition the training data into *k* regions;
2. For each of the *m* regions, a sparse LS-SVM model is developed;
3. Extracting the most informative support vectors from the *m* regions and merging them in one data pool;
4. Develop one global model based on the extracted support vectors.

These steps are illustrated in the following pseudo-code as implemented:


ApplyDataPartitioningAlgorithm(trainingData, k)\\
   % Applies a data partitioning algorithm to partition the training
   % data into k regions using Kmeans clustering in this case\\
   partitionedData = KMeansClustering(trainingData, k)\\
   return partitionedData
   
SparseLS_SVMModel(region)
   % Develops a sparse LS-SVM model for the given region
   % code for developing a sparse LS-SVM model goes here
   model=Initiate_SparseLS_SVM(region)
   model=Tune_SparseLS_SVM(region)
   model=Train_SparseLS_SVM(region)
   return model
   
ExtractMostInformativeSupportVectors(partitionedData)
   % Extracts the most informative support vectors from 
   % all the regions
   supportVectors = emptyList()
   for each region in partitionedData do
    informativeSupportVectors = GetInformativeSupportVectors(region)
    supportVectors = supportVectors + informativeSupportVectors
   return supportVectors
   
DevelopGlobalModel(supportVectors)
   % Develops a global model based on the extracted support vectors
   % code for developing a global model goes here
   model=Initiate_LS_SVM(supportVectors)
   model=Tune_LS_SVM(supportVectors)
   model=Train_LS_SVM(supportVectors)
   return model


It is worth mentioning that this approach can be extended to a multi-layer approach depending on the local and total data size.

## 3 Simulation experiments

**Fig 3:**
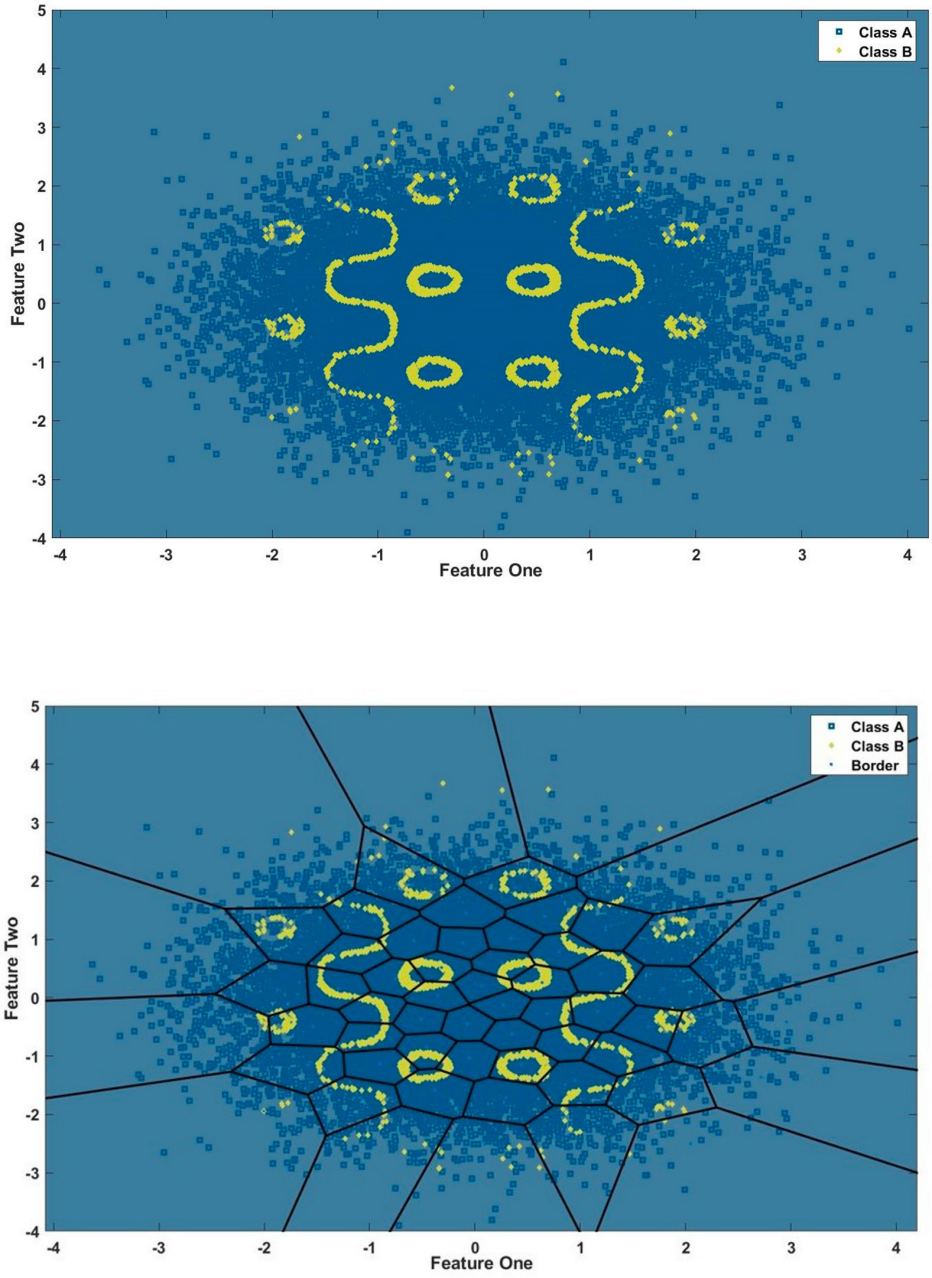


This section investigates the GLocal-LS-SVM algorithm by applying it to a synthetic dataset and comparing its performance to the LS-SVM model. The generated synthetic dataset is designed considering several properties: nonlinearity, class imbalance (1:9), medium-size data (10,000 samples), and scattered individual patterns, as shown in Fig 3a. As shown in Fig 2, the complete training data (90%) is applied to a data partitioning algorithm such as the Kmeans algorithm. The resulting partitioned data using the Kmeans algorithm is shown in Fig 3b.

All experiments in this study are carried out using a system Type x64-based PC, Processor Intel(R) Core(TM) i7-6820HQ CPU @ 2.70GHz, 2701 MHz, 4 Core(s), 8 Logical Processor(s), Installed Physical Memory (RAM) 8,00 GB). Moreover, MATLAB 2022a is used to implement the GLocal-LS-SVM algorithm and the comparative algorithms. More specifically, the toolbox of LS-SVMlab [17], the standard SVM toolbox of MATLAB, and the k-means clustering standard function of MATLAB.

### 3.1 Classification performance

**Fig 4:**
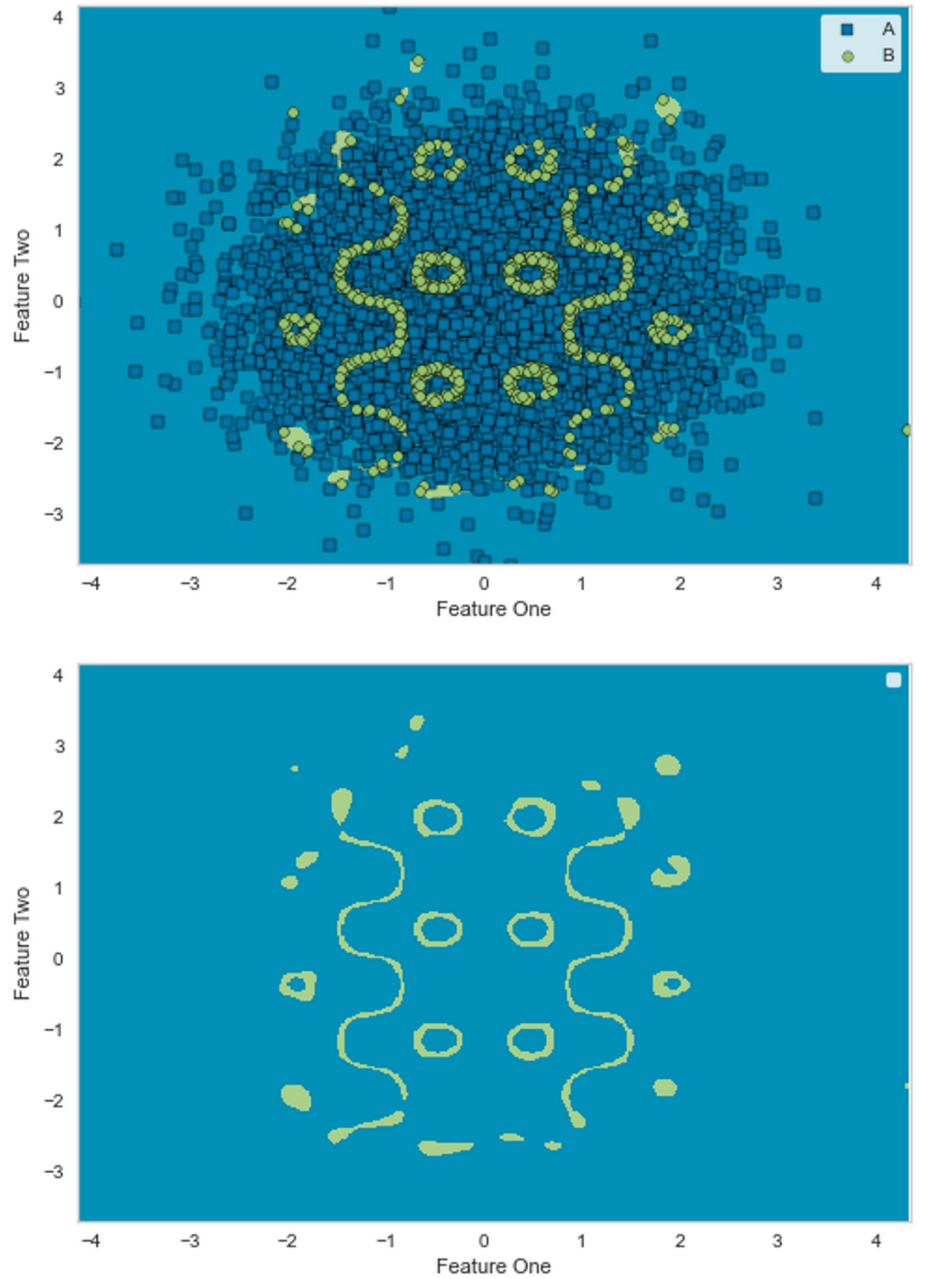


The reference classification performance for this synthetic dataset is the classification performance of the global LS-SVM classifier. The dataset is randomly shuffled for ten rounds and split every round into training and test subsets with percentages 90% and 10% respectively for the whole experiment. Firstly, the developed global LS-SVM classifier is trained and visualised as shown in Fig 4. In Fig 4a, the classifier is visualised in combination with the training dataset in the input space. Finally, in Fig 4b, the classifier is depicted in the input space without the training dataset for clear visualisation.

**Fig 5:**
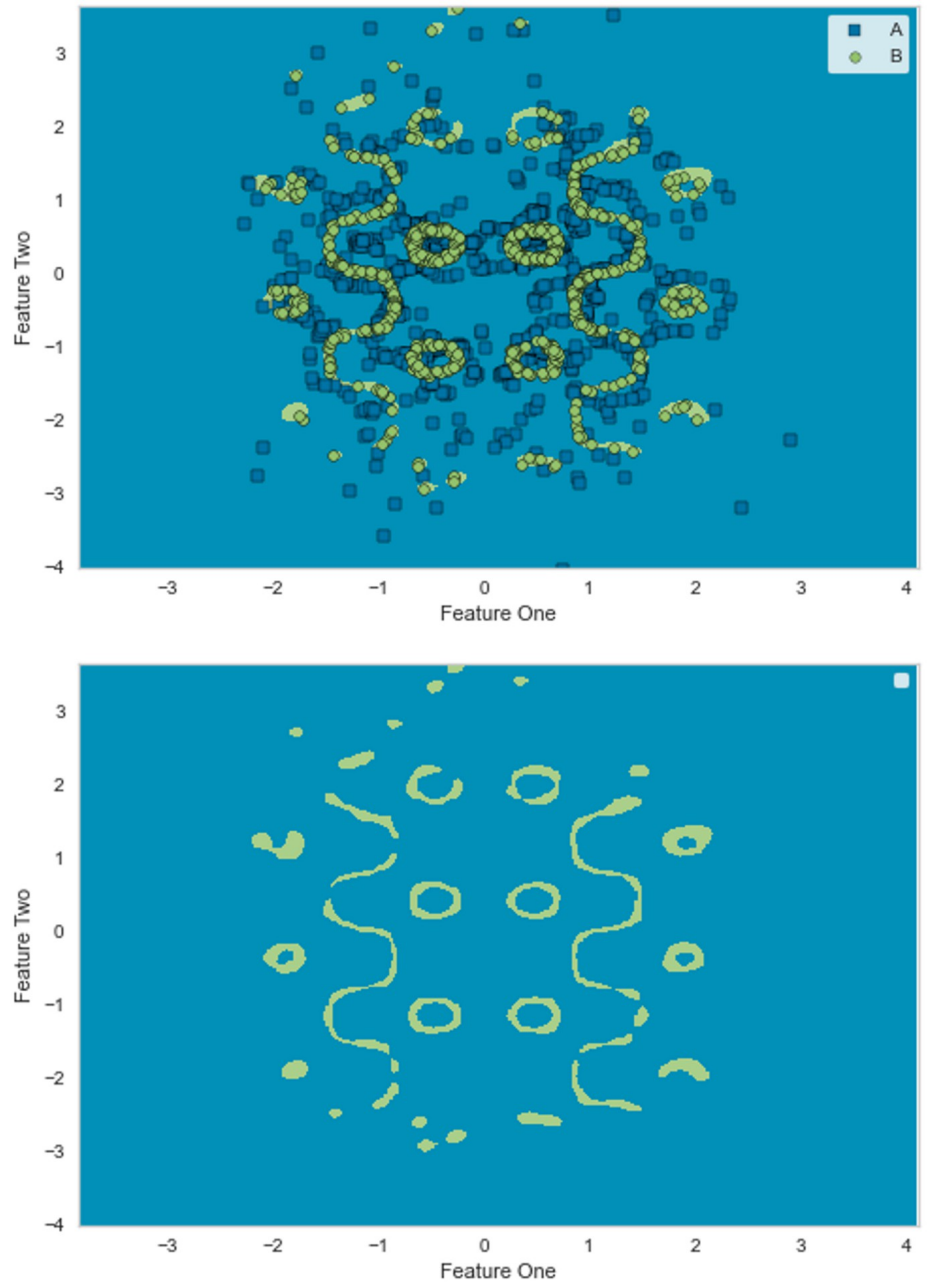


The GLocal-LS-SVM model is developed based on partitioning the training dataset into many partitions. In this experiment, the dataset is partitioned into 100, 90, 80, 70, 60, 50, and 40 data partitions to investigate the influence of the number of partitions on the error and the time performance. For instance, in Fig 5a, the resulting model of the GLocal-LS-SVM (90 partitions) is depicted in combination with the reduced training data (predetermined local support vectors). In Fig 5b, the GLocal-LS-SVM model is depicted in the original input space without the reduced training data. In Table 1, both sensitivity and selectivity of the standard LS-SVM and GLocal-LS-SVM models are depicted. Both sensitivity and selectivity computations are done considering the positive class is the minority class (class B).

**Table 1:** Comparing the classification performance of the global LS-SVM model to the GLocal-LS-SVM models (40-100 data-Partitions).

	LS-SVM	GLocal-LS-SVM 100-Partitions	GLocal-LS-SVM 90-Partitions	GLocal-LS-SVM 80-Partitions	GLocal-LS-SVM 70-Partitions	GLocal-LS-SVM 60-Partitions	GLocal-LS-SVM 50-Partitions	GLocal-LS-SVM 40-Partitions
Senstivity (%)	75.7 ± 3.2	75.4 ± 4.3	85.8 ± 2.3	84.1 ± 3.6	82.4 ± 1.9	80.2 ± 5.1	72.6 ± 4.8	68.8 ± 6.3
Selectivity (%)	98.1 ± 0.9	96.6 ± 1.3	97.4 ± 0.8	96.5 ± 1.6	99.3 ± 0.3	97.2 ± 1.1	98.6 ± 0.7	97.4 ± 1.5

For further investigation, in order to show the time performance advantage of the LS-SVM and its GLocal version, the GLocal-SVM and the standard SVM are applied to the same dataset. The classifcation performance of the Standard SVM and the GLocal-SVM with 40–100 partitions is depicted in Table 2. By applying the t-test with p-value 0.05 to test the significance between the error performance of the GLocal-LS-SVM with the different partitions and that of the GLocal-SVM, it is reported that the resulting error metrics are not significant for all partitions. Moreover, the t-test has shown significance between the LS-SVM model and the GLocal-LS-SVM with partitions 40–90 for sensitivity. However, no significance is reported for selectivity.

**Table 2:** Comparing the classification performance of the standard SVM model to the Glocal-SVM models (40-100 data-Partitions).

	Standard SVM	Glocal-SVM 100-Partitions	Glocal-SVM 90-Partitions	Glocal-SVM 80-Partitions	Glocal-SVM 70-Partitions	Glocal-SVM 60-Partitions	Glocal-SVM 50-Partitions	Glocal-SVM 40-Partitions
Sensetivity	76.1 ± 2.1	74.5 ± 3.6	82.8 ± 4.4	85.2 ± 2.7	83.1 ± 1.4	79.4 ± 4.2	74.3 ± 3.5	65.9 ± 5.2
Selectivity	98.5 ± 0.3	97.0 ± 0.9	98.17 ± 0.2	96.52 ± 1.3	97.1 ± 1.1	98.32 ± 0.2	95.56 ± 1.6	98.36 ± 0.5

### 3.2 Time performance

After showing the classification performance of the different models, the time performance of the LS-SVM and models is depicted in Table 3. In addition, the time performance of the GLocal-SVM and the standard SVM is shown in Table 4.

**Table 3:** Comparing the average time performance, in seconds, of the global LS-SVM model to the GLocal-LS-SVM models (100-40 Partitions), in addition to comparing the number of the average data points used to train the general model.

Model	LS-SVM	GLocal-LS-SVM (100 Partitions)	GLocal-LS-SVM (90 Partitions)	GLocal-LS-SVM (80 Partitions)	GLocal-LS-SVM (70 Partitions)	GLocal-LS-SVM (60 Partitions)	GLocal-LS-SVM (50 Partitions)	GLocal-LS-SVM (40 Partitions)
Time (Seconds)								
Accumulated Local Models Time	–	26.62	27.62	30.75	33.6	34.78	38.04	45.96
Average Local Model Time	–	0.26	0.32	0.41	0.49	0.57	0.79	1.2
General Model Time	3,266	36.24	34.32	34.17	34.40	32.41	30.14	31.68
Total Time	3,266	63.12	61.94	64.92	68.0	67.19	68.18	77.64
# Data points to Train General Model	9,000	1,611	1,634	1,662	1,622	1,566	1,574	1,526

**Table 4:** Comparing the time performance, in seconds, of the standard SVM model to the Glocal-SVM models (40-100 Partitions).

Model	Standard SVM	Glocal-SVM (100 Partitions)	Glocal-SVM (90 Partitions)	Glocal-SVM (80 Partitions)	Glocal-SVM (70 Partitions)	Glocal-SVM (60 Partitions)	Glocal-SVM (50 Partitions)	Glocal-SVM (40 Partitions)
Time (Seconds)								
Local Models Time	–	1,878	1,889	1,805	1,697	1,589	1,448	1,356
Average Local Model Time	–	18.78	20.98	22.56	24.24	26.48	28.96	33.90
Global Model Time	12,530	910	856	642	707	676	984	867
Total Time	12,530	3,188	2,745	2,447	2,404	2,265	2,432	2,230
# Data points to Train General Model	9,000	1,532	1,495	1,343	1,311	1,427	1,574	1,526

## 4 Real-world data

In this section, three publicly available real-world data sets are used to assess the performance of the GLocal-LS-SVM model.

The **Breast Cancer Wisconsin (diagnostic)** dataset [18] comprises a set of features that are obtained from digitised images of a fine needle aspirate (FNA) of breast masses. These features describe the characteristics of the cell nuclei present in the image. Each data sample represents an individual patient of 569 patients in this dataset. Moreover, each sample is labelled with benign or malignant breast mass diagnosis with an approximately 60: 40 label ratio.

The **Pima Indians Diabetes** dataset [19] is collected to predict the diabetes diagnosis of a set of subjects given specific diagnostic measurements. All patients are females at least 21 years old of Pima Indian heritage for this dataset. This dataset comprises 768 records, each of different subjects with eight features for each sample. Out of 768 subjects, 268 (34%) subjects are positively diagnosed with diabetes, and the rest are diagnosed with negative (66%); hence, the class ratio is approximately 1: 2.

The **Daphnet FoG** dataset [20] comprises labelled readings of 3 accelerometers attached to Parkinson’s disease patients who experience freezing of gait (FoG) during walking tasks. Since freezing of gait occurs rarely compared to other movement activities, the data is remarkably imbalanced since only 1/9 of all recorded instances correspond to the freezing class. Sensors were attached to three spots: the shank (just above the ankle) and the thigh (just above the knee) using an elasticised strap and Velcro. A third sensor was attached to the lower back via a belt. The number of patients in this study is 10. The sampling rate of the accelerometer recordings was 64 Hz, and the total number of the acquired samples was 1, 917, 887 within approximately 8.32 recording hours resulting to 4196 data points. The features were extracted from non-overlapping sliding windows of length 4 seconds. The extracted features are determined based on the state-of-the-art and comparative studies [7, 21]. These features are: interquartile range, amplitude kurtosis, root mean square, variance, mean, standard deviation, skewness, minimum, median, maximum, mean-cross, and zero-cross.

### 4.1 Classification and time performance

In order to assess the classification performance of the investigated algorithms, the three datasets are randomly shuffled for ten rounds and split into training and test subsets with percentages 90 & 10% respectively.

For the **Breast cancer Wisconsin** dataset, the classification performance of the GLocal-LS-SVM model of 7 partitions is compared to those of the LS-SVM, GLocal-SVM (with 7 partitions), and SVM models which are the state-of-the-art models [3, 22]. In Table 5, the classification performance is depicted in terms of accuracy, *F*_1_-score for both benign and malignant classes. In addition, in Table 6, the time performance of the four models (GLocal-LS-SVM, LS-SVM, GLocal-SVM, and SVM) is depicted in terms of seconds reflecting the average elapsed time to develop the local models, accumulated loacl models, global models, and total the modelling time for each approach.

**Table 5:** Comparing the average error performance of the GLocal LS-SVM, and LS-SVM applied to the breast cancer Wisconsin (diagnostic) dataset.

	GLocal LS-SVM	LS-SVM	GLocal SVM	SVM
Accuracy (%)	97.0±1.2	97.06±1.5	96.7±0.9	97.5±1.1
*F*_1_-score (Benign)	0.967±0.013	0.975±0.010	0.958±0.021	0.967±0.032
*F*_1_-score (Malignant)	0.927±0.12	0.902±0.21	0.919±0.18	0.921±0.17

**Table 6:** Comparing the average time performance, in seconds, of the GLocal-LS-SVM model to the global LS-SVM model, Glocal-SVM, and standard SVM applied to the breast cancer Wisconsin (diagnostic) dataset.

Model	GLocal-LS-SVM	LS-SVM	GLocal-SVM	Standard SVM
Time (Seconds)				
Accumulated Local Models Time	1.75	–	22.61	–
Average Local Model Time	0.25	–	3.23	–
General Model Time	2.4	8.1	18.14	45.78
Total Time	4.15	8.1	40.75	45.78

Similarly, for the **Pima Indians diabetes** dataset, the classification performance of the GLocal-LS-SVM (with 10 partitions) model is compared to those of the LS-SVM, GLocal-SVM (10 partitions), and SVM. The classification performance of the two models is depicted in terms of accuracy, *F*_1_-score for both positive and negative diagnoses as shown in Table 7. In addition, in Table 8, the time performance of the four models (GLocal-LS-SVM, LS-SVM, GLocal-SVM, and SVM) is depicted in terms of seconds reflecting the average elapsed time to develop the local models, accumulated loacl models, global models, and total the modelling time for each approach.

**Table 7:** Comparing the average error performance of the GLocal-LS-SVM and LS-SVM applied to the Pima Indians Diabetes dataset.

	GLocal LS-SVM	LS-SVM	GLocal SVM	SVM
Accuracy (%)	80.7±2.4	79.5±3.2	80.52±1.6	80.08±2.8
F1-score (-ve)	0.855±0.035	0.829±0.028	0.845±0.025	0.854±0.021
F1-score (+ve)	0.726±0.041	0.702±0.031	0.738±0.032	0.692±0.025

**Table 8:** Comparing the average time performance, in seconds, of the GLocal-LS-SVM model to the global LS-SVM model, Glocal-SVM, and standard SVM applied to the Pima Indians diabetes dataset.

Model	GLocal-LS-SVM	LS-SVM	GLocal-SVM	Standard SVM
Time (Seconds)				
Accumulated Local Models Time	2.70	–	35.10	–
Average Local Model Time	0.27	–	3.51	–
General Model Time	3.2	15.54	20.13	59.64
Total Time	5.90	15.54	55.23	59.64

For **Daphnet FoG** dataset, the classification performance of the GLocal-LS-SVM model is evaluated using sensitivity, precision, and *F*_1_-score. Moreover, the classification performance of the GLocal-LS-SVM (with 30 partitions) is compared to those of the LS-SVM model, GLocal-SVM (with 30 partitions), and SVM in addition to best performing state-of-the-art models for this dataset, namely, the deep learning approach (Ravi2017), kNN-SVM, and kNN-LS-SVM. The comparative methods are chosen to be global and localised models to allocate the performance of GLocal-LS-SVM between them. The experiment setup of GLocal-LS-SVM and LS-SVM is similar to that of the state-of-the-art [7]. In Table 9, the sensitivity, precision, and *F*_1_-score of the GLocal-LS-SVM model are compared to those of LS-SVM, deep learning approach (Ravi2017), kNN-SVM, and kNN-LS-SVM models. In addition, in Table 10, the time performance of the four models (GLocal-LS-SVM, LS-SVM, GLocal-SVM, and SVM) is depicted in terms of seconds reflecting the average elapsed time to develop the local models, accumulated loacl models, global models, and total the modelling time for each approach.

**Table 9:** Comparing the average error performance of the GLocal LS-SVM, LS-SVM, Ravi (2017), kNN-SVM, and kNN-LS-SVM models applied to the Daphnet FoG dataset.

	GLocal LS-SVM	LS-SVM	GLocal SVM	SVM	Ravi (2017)	kNN-SVM	LS-SVM
Sensetivity (%)	77.1±2.7	68.75±3.4	73.15±4.5	69.82±2.5	59.92±4.1	60.00±3.2	72.92±1.5
Precision (%)	71.54±3.5	75.5±2.4	76.03±2.3	72.85±3.5	67.89±4.2	72.00±3.1	77.55±3.4
*F*_1_-score	0.745±0.013	0.719±0.022	0.746±0.017	0.713±0.031	0.637±0.052	0.655±0.023	0.752±0.020

**Table 10:** Comparing the average time performance, in seconds, of the GLocal-LS-SVM model to the global LS-SVM model, Glocal-SVM, and standard SVM applied to the Daphnet FoG dataset.

Model	GLocal-LS-SVM	LS-SVM	GLocal-SVM	Standard SVM
Time (Seconds)				
Accumulated Local Models Time	17.10	–	201.60	–
Average Local Model Time	0.57	–	6.72	–
General Model Time	26.21	376.85	52.35	1,106.42
Total Time	43.31	376.85	253.95	1,106.42

## 5 Discussion

As shown in the simulation experiments, the generated synthetic classification dataset is used to assess the performance of the GLocal-LS-SVM model. The different experimental setups (40-100 data partitions) of the GLocal-LS-SVM show comparable and superior classification performance to the global LS-SVM, especially for the sensitivity to predict the minority class. The outperformance of the GLocal-LS-SVM sensitivity reaches 10% more than that of the LS-SVM for the setup of 90 data partitions. At the same time, the time performance of the GLocal-LS-SVM models is significantly better than that of LS-SVM since the training time of the 90-Partitions GLocal-LS-SVM is approximately 1/50 of the training time of the LS-SVM Global model. Moreover, as shown in Fig 5a and 5b, it is evident that the required number of data points to train a similar or superior general model can decrease. More specifically, for the case of 90 data partitions (which provided the best error performance), the number of the training data points was reduced from 9, 000 to 1, 634 data points. The selectivity values of the GLocal-LS-SVM models are comparable to that of the LS-SVM, with no observed significance.

As mentioned in the introduction, the main objective of developing GLocal-LS-SVM models is to extract the most informative data points (local support vectors) prior to developing the general model. Predetermining these data points is hypothesised to reduce the number of the required data points to train the global LS-SVM model. This hypothesis is validated through the aforementioned experiments of the GLocal-LS-SVM models since the reduced number of data points over the different GLocal-LS-SVM models (40-100 Partitions) is 1600 ± 42 out of 9, 000 training data points which represent approximately 17.5%. This reduction in the number of data points drastically affected the consumed time to develop the GLocal-LS-SVM models compared to the global LS-SVM model, as shown in Table 3. The consumed time to train the LS-SVM model using the whole training set (9000 data points) is 3, 266 seconds. In contrast, the average training time of the GLocal-LS-SVM is approximately 67 seconds, representing 2% of that of the LS-SVM. Moreover, the time performance of the GLocal-SVM and the standard SVM shows the advantage of using the LS-SVM instead of the SVM. More specifically, the elapsed time to train the standard SVM is approximately 4 times of that of LS-SVM. Moreover, the average elapsed time to develop the GLocal-SVM is approximately 45 times of that of GLocal-LS-SVM models.

For the **Breast cancer Wisconsin** dataset, as an example of an approximately balanced dataset. The classification performance of the GLocal-LS-SVM model is comparable to those of the LS-SVM, GLocal-SVM and SVM with no reported significance based on the t-test, as shown in Table 5. On the other hand, the time performance of the GLocal-LS-SVM is significantly better than that of the other models as shown in Table 6.

Similarly, for the **Pima Indians diabetes** dataset, the error performance of GLocal-LS-SVM is comparable to those of the LS-SVM, GLocal-SVM and the SVM with no reported significance based on the t-test. However, the time performance of the GLocal-LS-SVM is significantly better than that of the other models.

For the **Daphnet FoG** dataset, the GLocal-LS-SVM model is assessed compared to the state-of-the-art techniques. These techniques comprise global learning algorithms (i.e. LS-SVM, deep learning (Ravi2017)) and localised learning algorithms (i.e. kNN-SVM and kNN-LS-SVM). As shown in Table 9, the classification performance of the GLocal-LS-SVM is comparable to the best-performing model (i.e. kNN-LS-SVM) and significantly better than the LS-SVM model (based on t-test with p-value 0.01). This result indicates that the GLocal-LS-SVM approach can provide a general model comparable/superior to any global learning approach. Moreover, GLocal-LS-SVM can provide high performance in challenging situations such as class imbalance, which global models cannot handle efficiently in contrast with localised learning models. Another critical remark is that the merged support vectors comprise more class-balanced data points than the original class-imbalanced dataset. For instance, the class-imbalance of **Daphnet FoG** dataset is improved from a 1: 9 imbalance ratio to approximately 1: 2 after extracting only the support vectors. Ultimately, in Table 10 the GLocal-LS-SVM in comparison with those of the models (i.e. LS-SVM, GLocal-SVM, and SVM) shows a significantly better time performance.

It is noteworthy that the GLocal approach effectively handled the class imbalance observed in both the synthetic and **Daphnet FoG** datasets compared to the standard LS-SVM. This finding suggests that GLocal-LS-SVM inherits the ability of the localised LS-SVM algorithm to tackle input-space challenges such as class imbalance.

## 6 Conclusions

Based on the obtained results from the synthetic and the real-world datasets, the proposed GLocal-LS-SVM algorithm shows several strong points. Firstly, the GLocal-LS-SVM model can provide a comparable classification performance to the global models and the GLocal version of the standard SVM with significantly less computational complexity for the different datasets with sizes ranging between 569 and 10, 000 data points. Secondly, the time performance showed the significant advantage of using the LS-SVM instead of SVM as hypothesised earlier. Thirdly, the acceptable performance for the unbalanced dataset proves the previously mentioned hypothesis that the GLocal-LS-SVM algorithm can provide a general model that considers the dataset’s local characteristics, which is an inherited property from localised learning.

From an application perspective, the GLocal-LS-SVM algorithm allows learning models locally on distributed data sources, which supports a version of federated learning without transferring all data to a central data pool to train the general model. This version of federated learning may require additive privacy-preserving procedures once the shared support vectors are at a re-identification risk for sensitive data. However, having a sample of the data in the input space allows feature selection at the server considering all data sources at once. Furthermore, this version of federated learning does not require a cyclical and iterative process, which is essential for conventional federated learning approaches. Moreover, it is worth mentioning that implementing federated learning with the GLocal-LS-SVM algorithm shall start directly from building the local models as the data is already partitioned over the different data sources. In addition, the GLocal-LS-SVM algorithm supports parallel computing in general as each local model can be developed on a separate edge/site.

In conclusion, this study introduces and investigates the novel GLocal-LS-SVM as a potential solution to the problems of decentralized modelling on distributed data sources which can support federated learning and edge machine learning. In addition, it can allow for simplifying the learning process from a large-sized dataset by identifying only the most informative data points. Moreover, GLocal-LS-SVM could inherit the capability of localized learning to capture the local data patterns in the input space which was proven by handling the class imbalance problem.

For future work, it is suggested to implement this algorithm on a physically distributed system and a federated learning setup in addition to evaluating the performance of the algorithm compared to training the model on the whole data samples. For the algorithm itself, we suggest integrating a privacy-preserving method into the GLocal-LS-SVM to assure the data privacy requirement for the federated learning approach.
